# GABAergic synapses from the ventral lateral septum to the paraventricular nucleus of hypothalamus modulate anxiety

**DOI:** 10.3389/fnins.2024.1337207

**Published:** 2024-03-19

**Authors:** Ying-Juan Liu, Yan Wang, Jiao-Wen Wu, Jie Zhou, Bai-Lin Song, Yi Jiang, Lai-Fu Li

**Affiliations:** Research Center of Henan Provincial Agricultural Biomass Resource Engineering and Technology, College of Life Science and Agriculture, Nanyang Normal University, Nanyang, China

**Keywords:** lateral septum, GABA, paraventricular nucleus of hypothalamus, anxiety, stress

## Abstract

Emotional disorders, such as anxiety and depression, represent a major societal problem; however, the underlying neurological mechanism remains unknown. The ventral lateral septum (LSv) is implicated in regulating processes related to mood and motivation. In this study, we found that LSv GABAergic neurons were significantly activated in mice experiencing chronic social defeat stress (CSDS) after exposure to a social stressor. We then controlled LSv GABAergic neuron activity using a chemogenetic approach. The results showed that although manipulation of LSv GABAergic neurons had little effect on anxiety-like behavioral performances, the activation of LSv GABAergic neurons during CSDS worsened social anxiety during a social interaction (SI) test. Moreover, LSv GABAergic neurons showed strong projections to the paraventricular nucleus (PVN) of the hypothalamus, which is a central hub for stress reactions. Remarkably, while activation of GABAergic LSv–PVN projections induced social anxiety under basal conditions, activation of this pathway during CSDS alleviated social anxiety during the SI test. On the other hand, the chemogenetic manipulation of LSv GABAergic neurons or LSv^GABA^–PVN projections had no significant effect on despair-like behavioral performance in the tail suspension test. Overall, LS GABAergic neurons, particularly the LSv GABAergic–PVN circuit, has a regulatory role in pathological anxiety and is thus a potential therapeutic target for the treatment of emotional disorders.

## 1 Introduction

In modern society, emotional or physical stress is ubiquitous. Although moderate levels of stress may improve fitness, excessive stress can disrupt body homeostasis and induce cardiovascular, metabolic, and immunological diseases and related mental disorders, such as anxiety and depression (Sandi and Haller, 2015). The etiology of excessive anxiety and depression has been intensively studied over the past few decades; however, the detailed molecular pathways involved remain largely unknown (Fries et al., 2023). Therefore, current medications are generally poorly targeted with highly variable efficacy (Brewster et al., 2023).

Several forebrain and limbic brain structures are involved in stress-induced psychiatric disorders, including but not limited to the medial prefrontal cortex, hippocampus, hypothalamus, amygdala, and nucleus accumbens (NAc) (McEwen and Morrison, 2013; Ebner and Singewald, 2017). The lateral septum (LS) is a forebrain structure that is functionally and anatomically related to the limbic system and has been implicated in the regulation of mood, motivation, fear, and various social functions (Wirtshafter and Wilson, 2021; Menon et al., 2022). Anatomically, the LS can be roughly divided into two main divisions, the dorsal and ventral subregions (LSd and LSv, respectively), which are distinct in terms of their receptors, connections, and functions (Rizzi-Wise and Wang, 2021). Although many LS-related studies do not differentiate between these divisions in describing their findings (Wirtshafter and Wilson, 2021), increasing evidence indicates that the LSv is more intimately involved in the regulation of emotional behaviors (Xu et al., 2019; Mu et al., 2020), whereas the LSd is more closely involved in mediating associations with reinforcements, such as cocaine use (McGlinchey and Aston-Jones, 2018; Pantazis and Aston-Jones, 2020). Our recent study also found that the reduced expression of metabotropic glutamate receptors 2 and 3 in the LSv is a phenotype of stress vulnerability in male mice (Wang et al., 2022). The LS primarily consists of GABAergic neurons and a few glutamatergic or cholinergic neurons (Zhao et al., 2013). Therefore, further investigations of the functions and roles of these two types of neurons involved in stress reactions in the LSv would be noteworthy.

The LS receives strong inputs from the hippocampus and shares bidirectional connections with the hypothalamus (Sheehan et al., 2004; Deng et al., 2019). A few studies have investigated LS pathways in stress induced emotional and behavioral disorders. For example, Wang et al. (2021) found that the LS to the dorsal periaqueductal gray circuit is involved in depression induced by chronic unpredictable stress. Li et al. (2023) found that the LS–anterior hypothalamic nucleus and the LS–NAc pathways are involved in social reward deficits induced by chronic social defeat stress (CSDS). Moreover, Mu et al. (2020) found that the hippocampal ventral subiculum to the LSv to the lateral hypothalamus tuberal nucleus circuit is involved in emotional stress-induced self-grooming. The paraventricular nucleus (PVN) of the hypothalamus is an important brain region for homeostatic regulation and coordinates numerous behavioral and physiological adaptations to stress (Gray et al., 2015). A bidirectional connection exists between the PVN and LS (Deng et al., 2019). Recently, Xu et al. (2019) found that the optogenetic activation of the PVN–LSv pathway promotes stress-related grooming and escape behaviors. Therefore, an investigation into the involvement of the LSv–PVN pathway in stress-induced emotional and behavioral disorders would provide interesting results.

In this study, we first investigated stress-reactive neurons in the LSv following CSDS and evaluated anxiety- and depression-related behaviors after the activation or suppression of LSv GABAergic neurons under both basal and stress conditions. Then, we studied the GABAergic neuronal pathways from the LSv to the PVN and determined the role of LSv^GABA^–PVN projections in stress-related behaviors.

## 2 Materials and methods

### 2.1 Animals

This study used male C57BL/6 (C57) mice (8–10 weeks) purchased from Aniphe BioLab (Nanjing, China) and Kunming mice (6–8 months) purchased from Huaxing Experimental Animal Farm (Zhengzhou, China). The animals were raised under standard conditions (22 ± 3°C, 12 h light/dark cycle, light on 7 a.m.). The experiments were performed in accordance with the Guide for the Care and Use of Laboratory Animals of China and approved by the Animal Care and Use Committee of Nanyang Normal University (approved no. 20211212002).

### 2.2 Viral vectors

Adeno-associated viruses (AAV) including AAV2/9-vGAT1-CRE-WPRE-PA (Cat#: PT-0346), AAV(Retro)-vGAT1-CRE-WPRE-PA (Cat#: PT-0347), AAV2/9-hSyn-Cre-WPRE-hGH-pA (Cat#: PT-0136), AAV2/9-EF1α-DIO-hM3D(Gq)-mCherry-WPREs-PA (Cat#: PT-0042), AAV2/9-EF1α-DIO-hM4D(Gi)-mCherry-WPREs-PA (Cat#: PT-0043), AAV2/9-EF1α-DIO-mCherry-WPREs-PA (Cat#: PT-0013), and AAV2/1-EF1a-DIO-eGFP-WPRE-hGH-PA (Cat#: PT-0795) were used in this study. All the viral vectors were purchased from BrainVTA^®^ (Wuhan, China). The titre were > 2 × 10^12^ particles per mL.

### 2.3 Stereotaxic surgery and viral vectors transfer

For surgery, mice were anesthetized with 1.5–3.0% isoflurane and placed in a stereotaxic instrument. The virus was delivered by using 33-gauge syringe needles (Hamilton) and the injection rates were set at 50 nL/min. Unless other indicated, virus was bilaterally injected for a total volume of 300 nL per side. Cholera Toxin Subunit B-555 (CTB-555, Cat#: CTB-02, BrainVTA^®^) was delivered unilaterally for a total volume of 600 nL. After each injection, the needle was left in the brain for another 5 min before being slowly withdrawn in order to prevent the virus from leaking out. The expression duration for viral vectors and CTB-555 were 4 weeks and 10 days, respectively. The corresponding coordinates from bregma were: LSv: +0.5 mm, ML: ± 0.6 mm, DV: −3.7 mm; PVN: −0.8 mm, ML: ± 0.2 mm, DV: −4.6 mm. Mice with “non-targeted” injections were not included in the statistical analysis.

### 2.4 Chemogenetic manipulation

Water-soluble clozapine-N-Oxide (CNO, BrainVTA, Cat#: CNO-01) was dissolved in saline at a concentration of 1 mg/mL and stored at −20°C. The animals were intraperitoneally injected with CNO at 1 mg/kg 30 min before the behavioral tests.

### 2.5 Immunohistochemistry

Mice were anesthetized by injection of 2% sodium pentobarbital and perfused transcardially with phosphate-buffered solution (PBS), followed by 4% paraformaldehyde. The brains were quickly removed, post-fixed in 4% paraformaldehyde for two days, and dehydrated in 15, and 30% freshly prepared sucrose solutions. The brains were then cut into 40-μm coronal sections using a cryostat (Leica^®^ CM1950) and mounted onto gelatin-coated glass slides. For immunostaining, the sections were first rinsed in PBS (3 × 5 min), permeabilized with 0.1% Triton X-100 (1 h), and blocked in 5% normal goat serum blocking solution at room temperature (1 h). Sections were then incubated overnight in primary antibodies at 4°C. After thoroughly washing in PBS (3 × 5 min), the sections were incubated in secondary antibodies for 1 h at room temperature. Sections were then washed in PBS (3 × 5 min) and cover-slipped with an aqueous mounting medium, which contained DAPI (S2110, Solarbio, China). Finally, the sections were imaged under a fluorescence microscope (Leica^®^ DMi 3000). Positive cells were counted bilaterally in three sections from each brain spaced every 200 μm using Image J software. Individual means were obtained by averaged the counts to minimize variability. For c-Fos/GABA double staining, the GABA primary antibody (rabbit anti-GABA; 1: 300, A2052, Sigma) was first incubated followed by c-Fos antibody (mouse anti-c-Fos; 1: 500, ab208942, Abcam, UK). The colocalization rate was calculated by dividing the number of double-labeled cells by the number of GABA-labeled cells. The other primary antibodies used in this study were as follows: rabbit anti-c-Fos (1:1000, ab222699, Abcam, UK) and mouse anti-GAD65/67 (1:50, sc-377145, Santa Cruz, USA). The second antibodies were: Alexa Fluor^®^ 488 goat anti-mouse IgG (1:300, 115545146, Jackson, USA), DyLight 488 conjugated goat anti-rabbit IgG (1:300, Boster, China), Alexa Fluor^®^ TRITC goat anti-rabbit IgG (1:300, 111025046, Jackson, USA) and Alexa Fluor^®^ TRITC goat anti-mouse IgG (1:300, 115025003, Jackson, USA).

### 2.6 CSDS

CSDS was performed in mice as previously described (Li et al., 2021). Kunming mice were screened for aggressive behavior based on their attack latency (≤ 30 s on three consecutive screening tests). The aggressive Kunming mice were raised in the social defeat cage (320 × 215 × 170 mm), which was divided by clear perforated acrylic glass separators. Then, a subject was introduced into the cage of an aggressor for 10 min every day for 10 consecutive days. After the interactions, the two parties were separated by the perforated separator for 24 h for continued sensory contact. The aggressors were rotated daily to avoid habitation. Unstressed controls were housed in similar cages, one on each side of compartments separated by the perforated acrylic glass separator. They were handled similarly but without exposure to the aggressive Kunming mice.

### 2.7 Behavioral tests

Generally, all tests were conducted once a day under dim light (approximately 9:00–12:00 h). The animals were habituated in the testing room for 30 min before testing. Between sessions, the apparatus was cleaned with 30% ethanol and dried with napkins.

#### 2.7.1 Open-field test

The open-field test (OFT) is frequently used to evaluate anxiety levels and movement ability in rodents (Li et al., 2022a). Briefly, the animals were individually placed in a plastic box (50 × 50 × 50 cm, length × width × height) for 5 min. The floor of the box was virtually divided into 16 equal square quadrants and the four central quadrants were designated the central area. The time spent in the central quadrants and the total distance traveled during the test were automatically recorded via a video tracking system (SuperMaze Systems, Shanghai Xinruan^®^, China).

#### 2.7.2 Elevated plus maze test

The elevated plus maze (EPM) apparatus consisted of two open arms (35 × 5 × 1 cm) and two closed arms (35 × 5 × 15 cm), which crossed in the middle to form an open center (5 × 5 cm). The maze was elevated to a height of 70 cm above the floor. Initially, an animal was placed in the open center facing an open arm and allowed to freely explore for 5 min. The total time spent in the open and closed arms was measured by a video-tracking system (SuperMaze Systems, Shanghai Xinruan^®^, China).

#### 2.7.3 Social interaction test

The social interaction (SI) test is an effective procedure to measure social anxiety in rodents (Zhang et al., 2021). The test was conducted in the same open-field box but with a wire mesh cage (10 × 10 cm) in the middle of one side of the box. The floor of the box was virtually divided into an interaction zone (around the wire mesh cage, 30 × 20 cm) and a surrounding zone. The test consisted of two trials of 5 min each. In the first trial (target absent), the wire mesh cage was empty, and the mice were allowed to freely explore the box. In the target trial, a novel Kunming male mouse was placed into a wire mesh cage. During both trials, the times spent in the interaction and surrounding zones were recorded with a digital video tracking system (SuperMaze Systems, Shanghai Xinruan^®^, China). The target mice were used for a maximum of two times per day. The SI ratio, time spent in the interaction zone of the target trial divided by the time spent in the interaction zone of the no-target trial, was calculated to evaluate sociability.

#### 2.7.4 Tail suspension test

The despair-like behavior of mice was analyzed with the tail suspension test (TST) just as previously conducted (Li et al., 2022b). Briefly, the mice were suspended individually by an adhesive tape placed 1 cm from the tip of the tail. The behavioral performances of the mice were recorded by a video recorder for 6 min. The immobility time during the last 5 min was measured by two trained observers who were blinded to the grouping.

### 2.8 Statistical analysis

Statistical analysis was conducted with SPSS software version 22.0. Differences between two individual groups were compared using two-tailed unpaired Student’s *t*-tests. For group comparisons, data were compared using one-way or two-way analyses of variance (ANOVA), or two-way repeated-measures ANOVA followed by Bonferroni *post hoc tests* when significant main effects or interaction effects were found. To confirm the significance and calculate the effect size, Bayesian *t*-test or Bayesian ANOVA was conducted using default priors (Keysers et al., 2020). Data are presented as mean ± SE and *p* values <0.05 were considered statistically significant. The statistical analysis and quantifications were presented in Supplementary material 1. The raw data are deposited in Mendeley Data.

## 3 Results

### 3.1 CSDS leads to increased activation of LSv GABAergic neurons after exposure to a stressor

**Figure 1:**
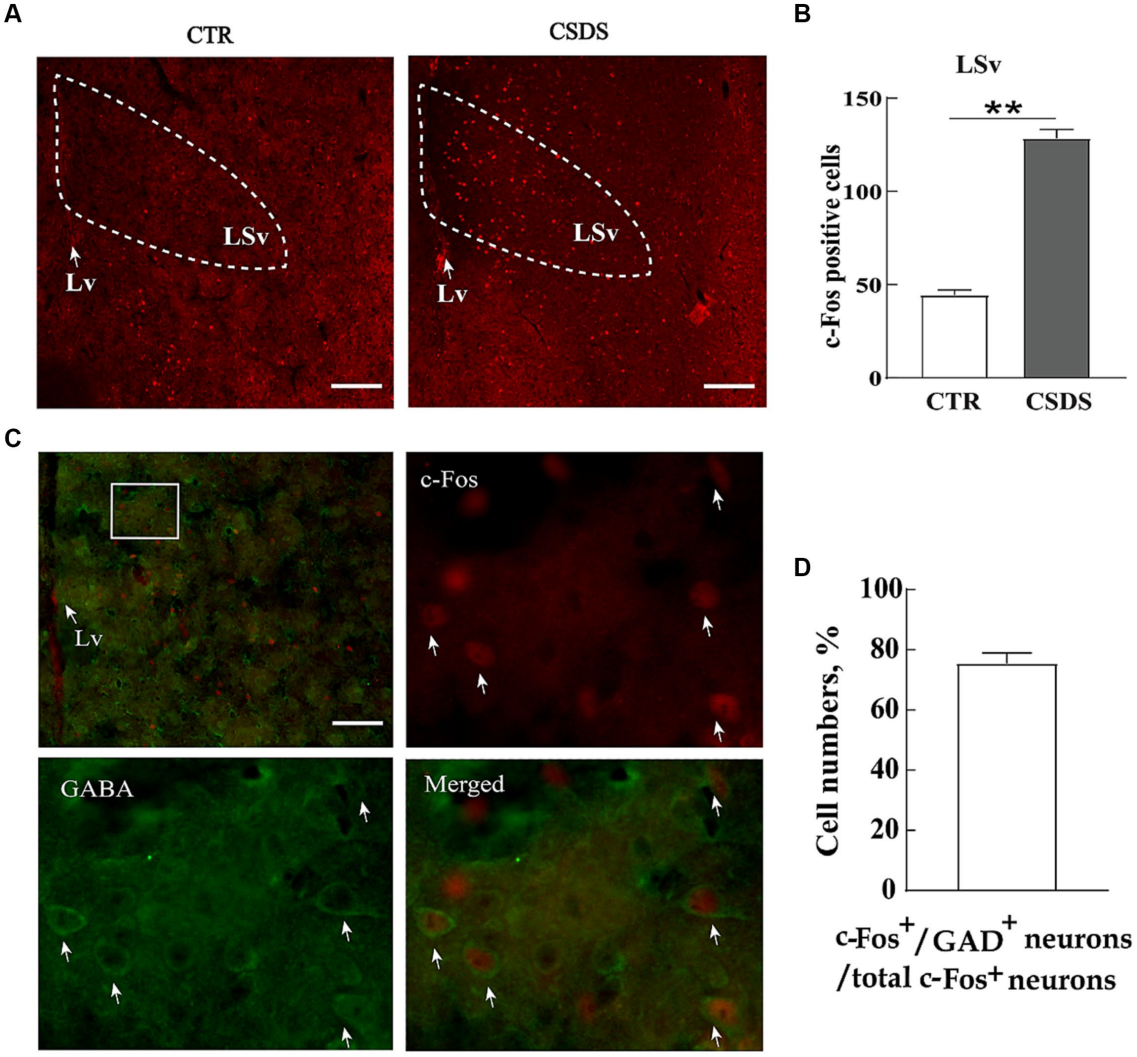
LSv GABAergic neurons are activated by CSDS. **(A,B)** Representative images and quantification of c-Fos positive cell numbers in the LSv. **(C)** Representative images of GABA (green) and c-Fos colocalization in the LSv. The white squares indicate the position where the other three images were taken. Arrows indicating co-localized cells. Scale bars = 100 μm. **(D)** GABA and c-Fos colocalization rates qualifications. Independent-sample *t*-tests, *n* = 7 animals in each group. Data are presented as mean ± SE; ^*^p < 0.05, ^**^p < 0.01. CTR, control; CSDS, chronic social defeat stress; LSv, ventral part of the lateral septum.

To identify whether neuronal activity in the LSv was associated with stress-induced emotional disorders, we compared the number of c-Fos immunoreactive neurons (a cellular marker of neural activity) in the LSv between CSDS and control mice after exposing them to a social stressor. The results showed a notable increase in c-Fos-positive neurons in the LSv of CSDS mice 90 min after exposure to trapped Kunming mice (Figures 1A,B; *t*_(12)_ = 16.504, *p* < 0.01, Cohen’s d = 8.822). Previous studies had indicated that the LS consisted primarily of GABAergic neurons (Zhao et al., 2013). In the following double immunofluorescence staining studies, we found about 75.4% ± 9.3% c-Fos positive cells were co-localized with GABA-labeled neurons (Figures 1C,D). Therefore, our following experiments were primarily focus on the roles of LSv GABAergic neurons on anxiety- and depression-like behavioral performances.

### 3.2 Chemogenetic manipulation of LSv GABAergic neurons did not significantly affect anxiety- and despair-like behavioral performances

**Figure 2:**
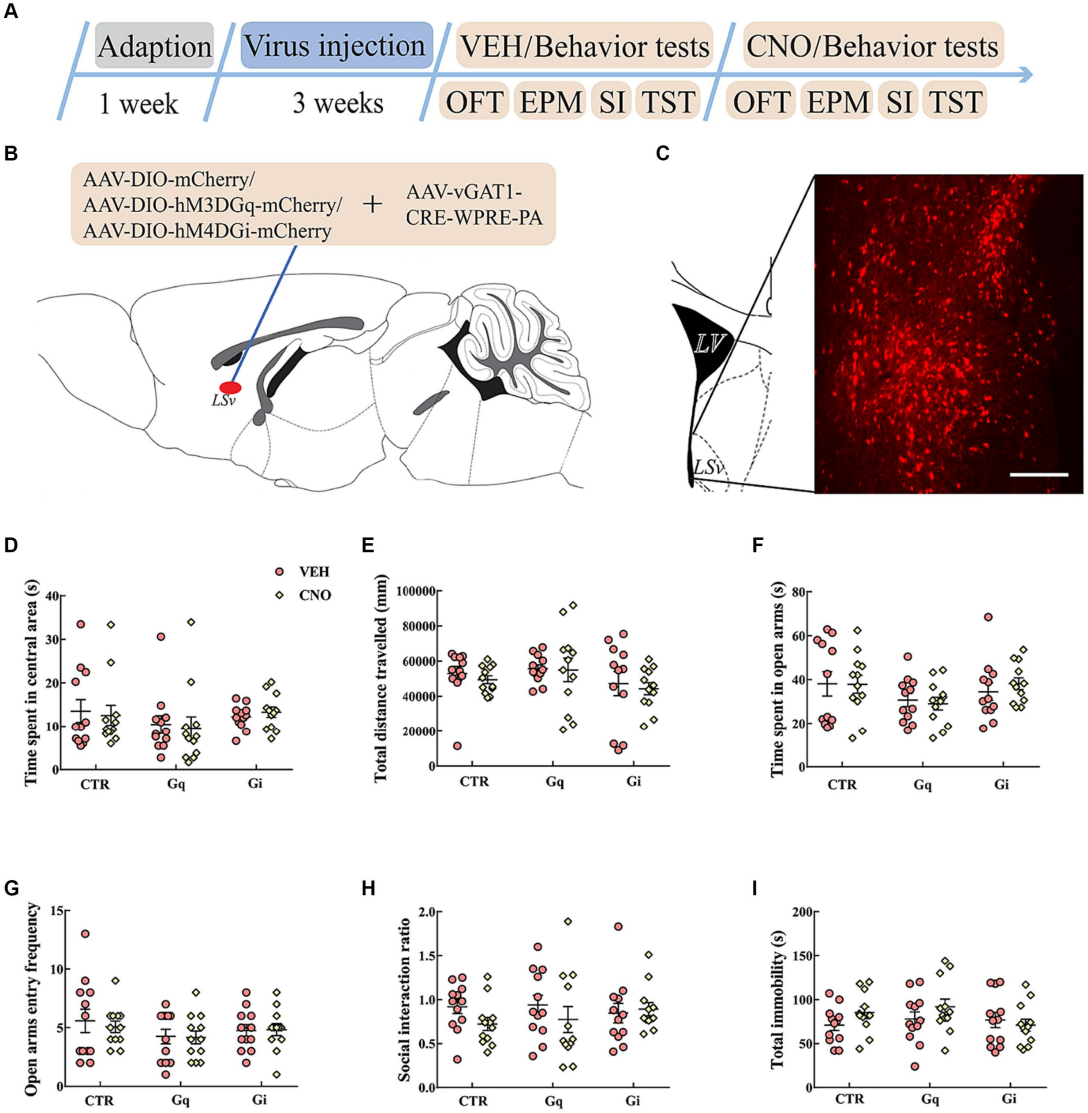
Chemogenetic modulation of LSv GABAergic neurons. **(A)** Timeline of experiments; **(B)** schematic of chemogenetical manipulations; **(C)** immunohistological image showing DREADDs expressions in the LSv, scale bar = 100 μm; **(D,E)** time spent in the central area and total distance traveled in the OFT; **(F,G)** time spent in the open arms and open arm entry frequencies in the EPM test; **(H)** SI ratio in the SI test; **(I)** total immobility time in the TST. Data are presented as mean ± SE and analyzed by two-way repeated measures ANOVA. *N* = 12 animals in each group; ^*^p < 0.05, ^**^p < 0.01. OFT, open-field test; EPM, elevated plus maze test; SI test, social interaction test; TST, tail suspension test; CTR, virus control.

To explore the causal role of LSv GABAergic neurons in anxiety- and depression-like behaviors, we chemogenetically manipulated LSv GABAergic neurons by injecting AAV-DIO-hM3D(Gq)-mCherry (Gq) + AAV-vGAT1-CRE, AAV-DIO-hM4D(Gi)-mCherry (Gi) + AAV-vGAT1-CRE, or AAV-DIO-mCherry (virus control) + AAV-vGAT1-CRE into the LSv (Figures 2A,B). Abundant DREADDs were expressed in the LSv (Figure 2C). Immunohistochemical staining revealed that approximately 72% of mCherry cells coexpressed with GAD65/67, whereas approximately 54% of GAD65/67^+^ cells coexpressed with mCherry (Supplementary Figure S1B). To verify the function of Gq/Gi, 1 mg/kg CNO was injected intraperitoneally. The neuronal active marker c-Fos was measured 90 min after the CNO injection. The results indicated that compared with the virus control groups, c-Fos–positive neurons were significantly elevated in the LSv of Gq animals and decreased in the LSv of Gi animals [Supplementary Figures S1C,D; *F*_(2,17)_ = 128.827, *p* < 0.01, *η*^2^ = 0.945; Gq vs. virus control and Gi vs. virus control, all *p* < 0.01]. The above results indicate the viability of this virus strategy.

Then we evaluated anxiety- and despair-like behavioral performances following CNO injections. The results indicated that the chemogenetic manipulation of LSv GABAergic neurons did not significantly affect behavioral performances in the OFT (Figures 2D,E), EPM test (Figures 2F,G), SI test (Figure 2H), and TST (Figure 2I).

### 3.3 Chemogenetic activation of LSv GABAergic neurons during CSDS affected social anxiety

To assess the functions of LSv GABAergic neurons in chronic stress induced behavioral dysfunctions, LSv GABAergic neurons were manipulated during CSDS. All mice were given CNO or vehicle each day before defeat. We first examined the effects of CSDS and CNO in virus control animals. As expected, CSDS induced anxiety-like behavioral performances in both the OFT and SI test [time spent in central area in the OFT: group effect, *F*_(1, 20)_ = 13.533, *p* < 0.01, *η*^2^ = 0.363, BF_(incl)_ = 21.624, CSDS stress vs. non-stress, *p* < 0.01; Supplementary Figure S2B. SI ratio: F_(1, 20)_ = 29.843, *p* < 0.01, *η*^2^ = 0.559, BF_(incl)_ = 845.884; CSDS vs. non-stress, *p* < 0.01; Supplementary Figure S2F], but the results of the EPM test and TST were inconclusive (Supplementary Figures S2D,E,G). On the other hand, CNO treatment did not significantly affect anxiety- or despair-like behavioral performances in the virus control group.

**Figure 3:**
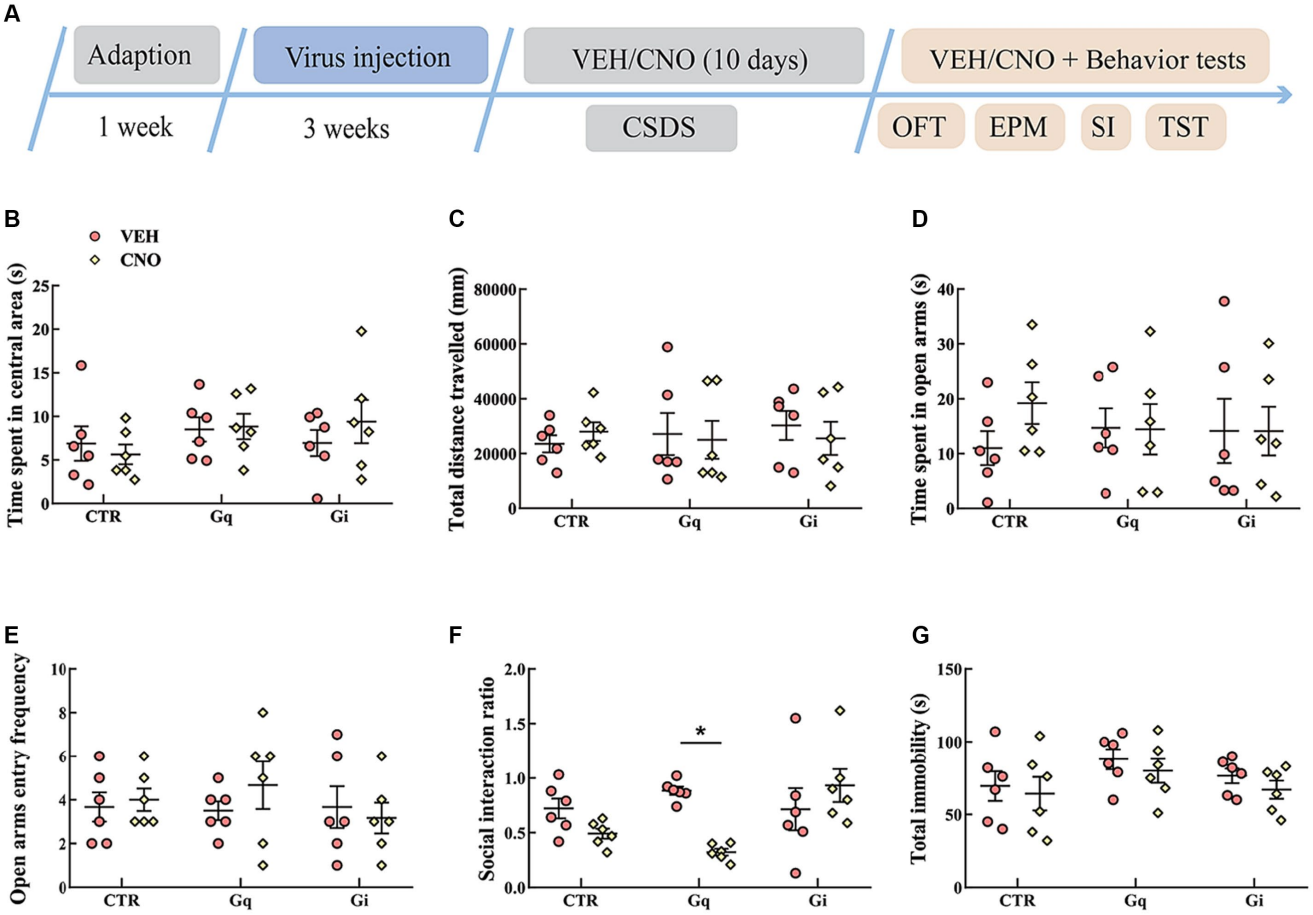
Chemogenetic modulation of LSv GABAergic neurons during CSDS. **(A)** Timeline of experiments; **(B,C)** time spent in the central area and total distance traveled in the OFT; **(D,E)** time spent in the open arms and open arm entry frequencies in the EPM test; **(F)** social interaction ratio in the SI test; **(G)** total immobility time in the TST. Data are presented as mean ± SE and analyzed by two-way ANOVA, *n* = 6 animals in each group. ^*^p < 0.05, ^**^p < 0.01. CTR, virus control; OFT, open-field test; EPM, elevated plus maze test; SI test, social interaction test; TST, tail suspension test; CTR, virus control.

The results of the activation or inhibition of LSv GABAergic neurons during CSDS were presented in Figure 3. In the SI test, a significant “treatment × group” interaction effect was observed [*F*_(2, 30)_ = 6.289, *p* < 0.01, *η*^2^ = 0.240, BF_(incl)_ = 9.710]. The following *post-hoc* comparation results showed that chemogenetic activation of LSv GABAergic neurons during CSDS significantly reduced the SI ratio (Gq: VEH vs. CNO, *p* = 0.017; Figure 3F). However, manipulation of LSv GABAergic neurons during CSDS did not significantly affect behavioral performances in the OFT, EPM test, and TST (Figures 3B–E,G).

### 3.4 Identification of neuronal projections from the LSv^GABA^ to the PVN

**Figure 4:**
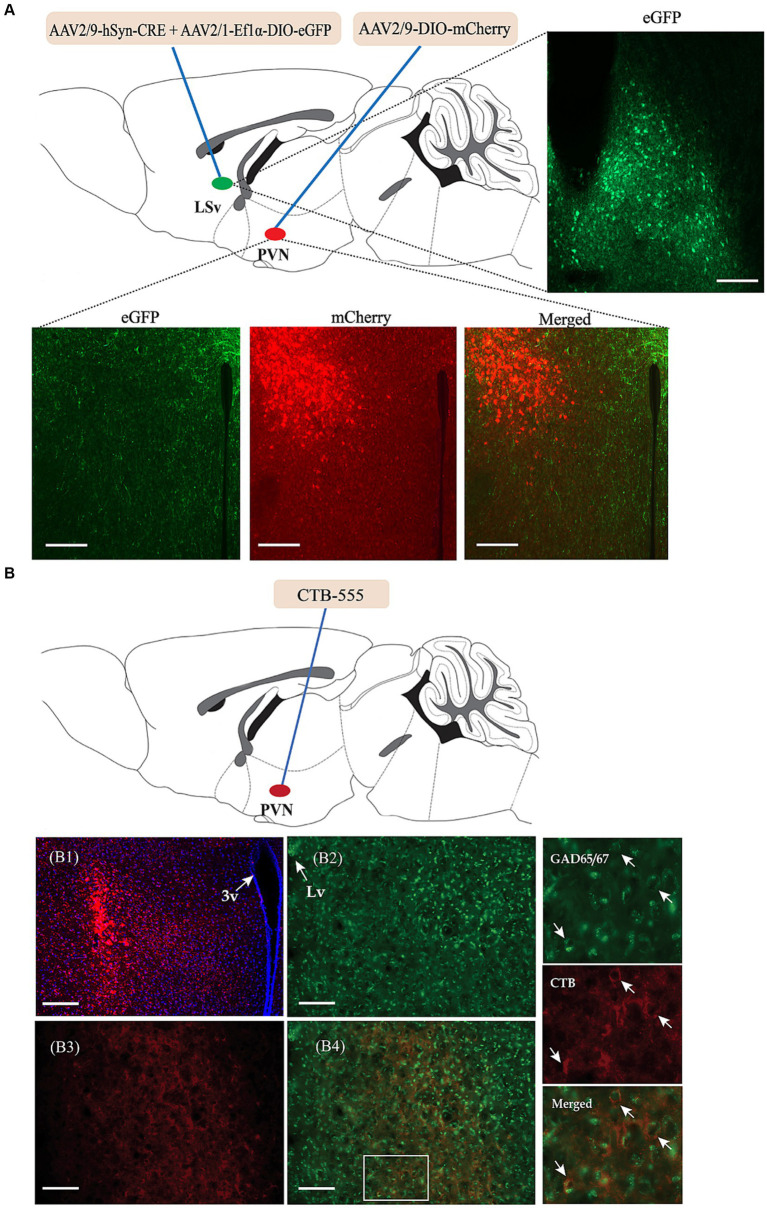
Identification of the neuronal projections from the LSv^GABA^ to the PVN. **(A)** Anterograde virus verification. Virus strategy: AAV2/9-hSyn-Cre + AAV-DIO-eGFP co-infused into the LSv; AAV-DIO-mCherry infused into the PVN. Representative images showing GFP-labeled neurons in the LSv, mCherry-labeled neurons and eGFP-labeled neural terminals in the PVN. **(B)** Retrograde tracer CTB-555 verification. **(B1)** Representative images showing the expression of CTB-555 in the PVN. **(B2,B3)** Representative images showing the expression of GAD65/67 and CTB in the LSv. **(B4)** Merged images of “**B2**” and “**B3**.” The right panels are enlarged images in the white box of “**B4**” showing colocalization of GAD65/67 neurons (green) and CTB (red) in the LSv. The arrows indicating double-labeled cells. Scale bars = 100 μm. *N* = 3 animals. LV, lateral ventricle; LSv, ventral part of the lateral septum; PVN, paraventricular nucleus of hypothalamus.

We then investigated the possible targeted brain regions influenced by the regulatory effect of GABAergic neurons in the LSv and focused primarily on the PVN. We first identified direct connections from the LSv to the PVN. As shown in Figure 4A, AAV2/9-hSyn-CRE + AAV-DIO-eGFP was injected into the LSv, whereas Cre-dependent AAV-DIO-mCherry was injected into the PVN. AAV2/9-hSyn-Cre, which enabled the Cre enzyme to spread anterogradely and monosynaptically into the soma of neurons in PVN (Wang et al., 2021), induced mCherry expression in the PVN. Consistently, abundance of mCherry-labeled neurons and eGFP labeled terminals were observed in both the PVN and peri-PVN four weeks later (Figure 4A). To further confirm LSv^GABA^–PVN projections, the retrograde tracer CTB-555 was injected into the PVN (Figure 4B), followed by immunofluorescence staining of the LSv sections with GAD65/67. The results showed a substantial number of GAD65/67+ (glutamate decarboxylase 65/67, a marker of GABAergic neurons) cells colocalized with CTB (Figure 4B) in the LSv, indicating LSv–PVN GABAergic projections.

### 3.5 Chemogenetic manipulation of LSv^GABA^–PVN projections influenced anxiety-like behaviors

**Figure 5:**
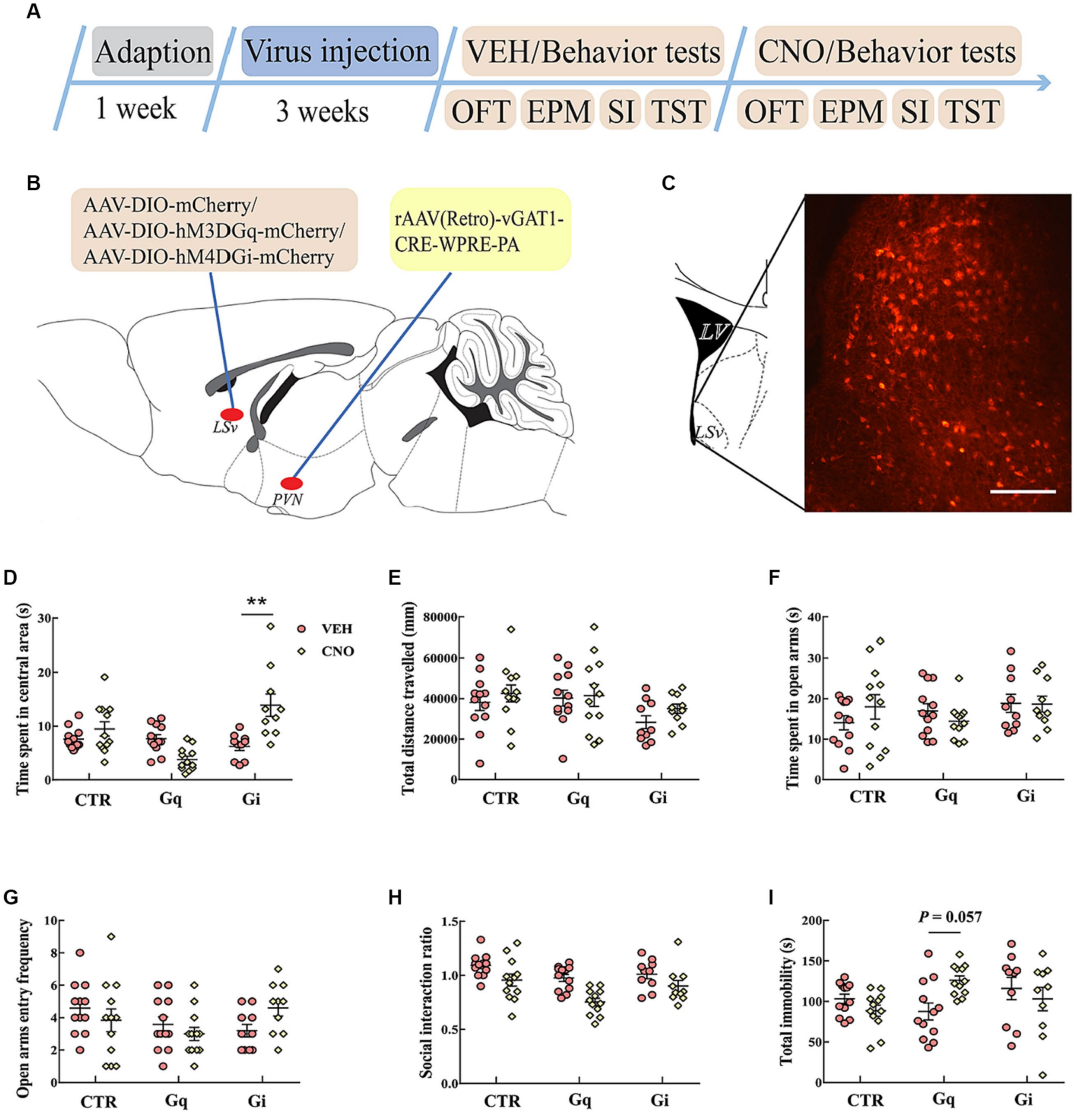
Chemogenetic modulation of LSv^GABA^-PVN projections. **(A)** Timeline of experiments; **(B)** schematic of chemogenetical manipulations; **(C)** immunohistological image showing DREADDs expressions in the LSv, scale bar = 100 μm; **(D,E)** time spent in the central area and total distance traveled in the OFT; **(F,G)** time spent in the open arms and open arm entry frequencies in the EPM test; **(H)** SI ratio in the SI test; **(I)** total immobility time in the TST. Data are presented as mean ± SE and analyzed by two-way repeated measures ANOVA. *N* = 10–12 animals in each group; ^**^p < 0.01. OFT, open-field test; EPM, elevated plus maze test; SI test, social interaction test; TST, tail suspension test; CTR, virus control.

To investigate the function of LSv^GABA^–PVN projections in anxiety and depression-like behaviors, AAV-DIO-hM3D(Gq)-mCherry, AAV-DIO-hM4D(Gi)-mCherry, or AAV-DIO-mCherry was injected into the LSv, whereas a retro-AAV containing the GAD1 promoter and Cre element (AAV(Retro)-vGAT1-Cre) was injected into the PVN (Figures 5A,B). Four weeks later, substantial mCherry-labeled cells were found in the LSv (Figure 5C). The following immunohistochemical staining results showed that approximately 83% of mCherry cells colocalized with GABA (Supplementary Figures S3A,B). We further found CNO treatment significantly reduced the c-Fos expression in the PVN in Gq-animals, which may partially indicate the GABAergic nature of these projections [Supplementary Figures S3C,D, *F*_(2,12)_ = 14.341, *p* < 0.01, *η*^2^ = 0.705, BF_(incl)_ = 44.854. Gq vs. Gi, *p* < 0.01; Gq vs. CTR, *p* = 0.042].

We then examined anxiety- and despair-like behavioral performances after manipulating these projections. In the OFT, two-way repeated measures ANOVA indicated an effect of “group” and “group × treatment” for the time spent in the central area [*F*_(2, 31)_ = 6.578, *p* = 0.004, *η*^2^ = 0.146, BF_(incl)_ = 3.257; *F*_(2, 31)_ = 16.761, *p* < 0.01, *η*^2^ = 0.244]. *Post hoc* comparison revealed that CNO-treated Gi mice spent more time in the central area (Figure 5D. Gi: VEH vs. Gi_CNO, *p* < 0.01). Both CNO treatment and virus did not significantly affect the total distance traveled in the OFT (Figure 5E). In the EPM test, the chemogenetic manipulation of LSv^GABA^–PVN projections did not significantly affect the time spent in open arms (Figure 5F) and open arm entry frequencies (Figure 5G). In the SI test, the data revealed a main effect of “group” [*F*_(2, 31)_ = 8.465, *p* = 0.001, *η*^2^ = 0.136, BF_(incl)_ = 5.755] and “treatment” [*F*_(1, 31)_ = 18.257, *p* < 0.001, *η*^2^ = 0.218, BF_(incl)_ = 3510.247] but not their interaction [*F*_(2, 31)_ = 1.102, *p* = 0.345, *η*^2^ = 0.026, BF_(incl)_ = 0.638]. CNO treatment significantly reduced the SI ratio (VEH vs. CNO, *p* < 0001), especially in the Gq group (Gq: VEH vs. CNO, *p* = 0.01; Gi: VEH vs. CNO, *p* = 1; CTR: VEH vs. CNO, *p* = 0.604). In the TST, the data showed an interaction effect of “group × treatment” for the total immobility time [*F*_(2, 31)_ = 5.869, *p* = 0.007, *η*^2^ = 0.137, BF_(incl)_ = 20.151]. Subsequent *post hoc* comparison showed that there was a trend for chemogenetic activation of LSv^GABA^–PVN projections increased the immobility time (Figure 5I; Gq: VEH vs. CNO, *p* = 0.057), but the findings were inconclusive.

### 3.6 Chemogenetic activation of LSv^GABA^–PVN projections during CSDS alleviated social anxiety

To assess whether LSv^GABA^–PVN projections are involved in chronic stress induced behavioral dysfunctions, LSv GABAergic neurons specifically projected to PVN were manipulated during CSDS. The virus strategies were the same as in Figure 5B. Before CSDS manipulations, all the mice were given CNO or vehicle each day. Unsurprisingly, CSDS induced anxiety- or despair-like behavioral performances in virus control animals (Supplementary Figures S2H–M).

**Figure 6:**
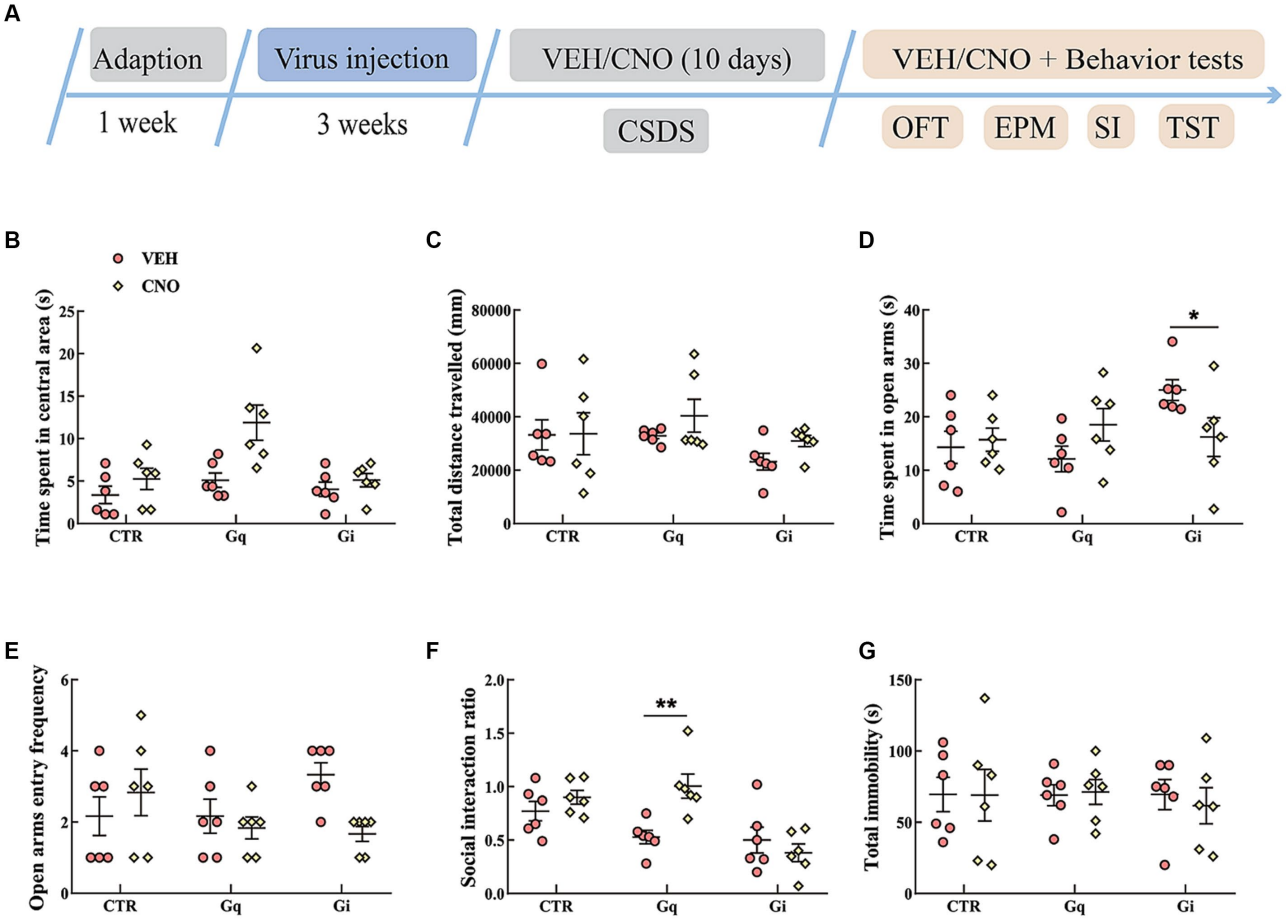
Chemogenetic modulation of LSv^GABA^-PVN projections during CSDS. **(A)** Timeline of experiments; **(B,C)** time spent in the central area and total distance traveled in the OFT; **(D,E)** time spent in the open arms and open arm entry frequencies in the EPM test; **(F)** SI ratio in the SI test; **(G)** total immobility time in the TST. Data are presented as mean ± SE and analyzed by two-way ANOVA. *N* = 6 animals in each group; data are presented as mean ± SE. ^*^p < 0.05, ^**^p < 0.01. CTR, virus control; EPM, elevated plus maze test; SI test, social interaction test; OFT, open-field test; TST, tail suspension test.

The results of the manipulation of LSv^GABA^–PVN projections during CSDS are presented in Figure 6. In the OFT, the data showed an effect of “group” [*F*_(2, 30)_ = 6.716, *p* = 0.004, *η*^2^ = 0.212, BF_(incl)_ = 5.904] and “treatment” [*F*_(1, 30)_ = 12.171, *p* = 0.002, *η*^2^ = 0.212, BF_(incl)_ = 10.844]. We also found a near significant effect of “group × treatment,” but the results were inconclusive [*F*_(2, 30)_ = 3.952, *p* = 0.030, *η*^2^ = 0.124, BF_(incl)_ = 2.376]. CNO treatment significantly increased the time spent in the central area in the OFT, especially in the Gq group (Figure 6B). Chemogenetic manipulation of LSv^GABA^–PVN projections did not significantly affect the total distance traveled in the OFT (Figure 6C). In the EPM test, the data indicated a “group × treatment” interaction effect [*F*_(2, 30)_ = 4.941, *p* = 0.014, *η*^2^ = 0.193, BF_(incl)_ = 4.312]. *Post hoc* comparison revealed that CNO-treated Gi mice spent less time in the open arms (Gi: VEH vs. CNO, *p* = 0.041; Figure 6D). For the SI ratio, the data showed a main effect of “group” [*F*_(2, 30)_ = 11.038, *p* < 0.001, *η*^2^ = 0.311, BF_(incl)_ = 23.957] and an interaction effect of “group × treatment” [*F*_(2, 30)_ = 6.466, *p* = 0.005, *η*^2^ = 0.182, BF_(incl)_ = 8.722]. Subsequent *post hoc* comparison revealed that chronic activation of LSv^GABA^–PVN projections significantly increased the SI ratio (Figure 6F; Gq_VEH vs. Gq_CNO, *p* < 0.01). Chronic manipulation of LSv^GABA^–PVN projections did not significantly affect the immobility time in the TST (Figure 6G).

## 4 Discussion

In this study, we explored the functions of LSv GABAergic neurons and their projections to the PVN in anxiety- and depression-like behavioral performances in male mice. Our main and novel findings are as follows: (1) CSDS increased c-Fos expression in LSv GABAergic neurons; (2) although direct manipulation of LSv GABAergic neurons had little effect on anxiety- and despair-like behavioral performances, the activation of LSv GABAergic neurons during CSDS aggravated social anxiety in Gq animals; (3) the LSv sent GABAergic projections to the PVN; and (4) the activation of LSv^GABA^–PVN projections under basal conditions induced social anxiety, whereas activation of this pathway during CSDS alleviated social anxiety. The above results indicated the LSv^GABA^-PVN projections played some regulatory role in anxiety-like behavioral performance in mice.

We found CSDS significantly elevated c-Fos expressions in the LSv (Figure 1B). Consistent with this, Mu et al. (2020) found robust c-Fos expression in the LSv after a body restrain stress in mice. In another study, Xu et al. (2019) reported that LSv neurons activities were rapidly increased following a sudden loud sound stressor. LSv neurons are predominantly GABAergic, but a few are glutamatergic or cholinergic (Vega-Quiroga et al., 2018). In this study, we found that GABAergic neurons were significantly activated in mice experiencing chronic social defeat stress (CSDS) after exposure to a social stressor (Figures 1C,D), which is consistent with the results of Wang et al. (2021) who induced chronic unpredictable stress in mice. Previous studies have indicated that LS GABAergic neurons are mostly calbindin- or calretinin-positive (Zhao et al., 2013). Further investigation should be conducted to identify the types of GABAergic neurons involved in stress.

In subsequent experiments, we found that direct manipulation of LSv GABAergic neurons did not significantly affect anxiety- or despair-like behavioral performance during the OFT, EPM test, SI test, and TST (Figure 2). However, Wang D. et al. (2023) found that the activation of LS GABAergic neurons induced anxiety during the OFT and EPM test. The authors further found that although the optogenetic activation of LS GABAergic adenosine A2A receptors (A2AR)-positive neurons did not significantly affect behavioral performances in the OFT and EPM test, it could significantly increase the immobility time in the TST (Wang M. et al., 2023). In another study, Mu et al. (2020) found that the optogenetic activation of LSv neurons induced robust self-grooming, which is frequently observed after stress. These conflicting results may be due to the manipulation of different types of neurons or subregions of the LS, which underscores the need to precisely delineate the special functions of specific subpopulations of LS neurons in future studies.

The chemogenetic activation of LSv GABAergic neurons during CSDS aggravated social anxiety in Gq animals during the SI test (Figure 3F). In a recent study, Li et al. (2023) found that the chemogenetic activation of LS neurotensin neurons, which are virtually GABAergic, reduced the SI ratio, social investigation, and social reward during a battery of social behavioral studies. Similarly, Wang et al. (2021) found that the activation of LS GABAergic neurons enhanced susceptibility to subchronic unpredictable stress. The above results indicate that LS GABAergic neurons are an important target for chronic stress reactions.

The LS is a major relay that connects the hippocampus with various subcortical regions, thus containing wide-range input–output projections (Rizzi-Wise and Wang, 2021; Besnard and Leroy, 2022). Using both anterograde and retrograde tracing strategies, we found direct GABAergic projections from the LSv to the PVN (Figure 4). This result is consistent with that of Herman et al. (2002). Subsequently, we found that chemogenetic manipulation of LSv^GABA^–PVN projections influences anxiety-like behavioral performance, i.e., inhibition increased the time spent in the central area during the OFT (Figure 5D), and activation reduced the SI ratio during the SI test (Figure 5H). However, both the activation and inhibition of LSv^GABA^–PVN projections did not significantly affect behavioral performances in the EPM test (Figures 5F,G). Inconsistent results on these behavioral paradigms have been frequently reported in previous studies, which may reflect the high variability and sensitivity of animal behaviors (Pentkowski et al., 2021; Stupart et al., 2022). Recently, Anthony et al. (2014) reported a disynaptic connection between the LS and PVN via the anterior hypothalamic nuclei, which are implicated in behavioral and hormonal anxiety. Similarly, Wang D. et al. (2023) found that the optogenetic activation of the LS^GABA^–lateral hypothalamus circuit induced anxiety and pain comorbidities. Mu et al. (2020) demonstrated that the optogenetic activation of the hippocampal ventral subiculum–LSv–lateral hypothalamus tuberal nucleus circuit triggered emotional stress. Thus, our study identified another LS-associated pathway involved in anxiety-like behavioral performance in mice. This raises the question of why the manipulation of LSv GABA neurons (Figure 2) and LSv^GABA^–PVN projections (Figure 5) get inconsistent results. One reasonable explanation may be that the manipulation of LSv GABA neurons may affect multiple neuronal projections, which then gains an integrated effect.

Another interesting finding of our study is that the chemogenetic activation of LSv^GABA^–PVN projections during CSDS alleviated social anxiety in Gq animals (Figure 6F), whereas the inhibition of this circuit increased anxiety during the EPM test (Figure 6D). The underlying neural mechanism for this effect remains unknown. Chronic manipulation of LSv^GABA^–PVN projections may trigger enduring changes in neural circuits, synapses, or neurotransmitters (Spitzer, 2017) or induce behavioral changes, such as self-soothing behaviors (grooming), which require further examination (Mu et al., 2020). It should be noted that although CSDS-exposed virus control animals showed a trend of reducing SI ratio in this experiment (Supplementary Figure S2L), the statistics were inconclusive [*F*_(1, 20)_ = 4.375, *p* = 0.049, *η*^2^ = 0.170, BF_(incl)_ = 1.749]. This perhaps due to the small sample size (*n* = 6 in each group), but may confound the above increased SI ratio result seen with Gq mice (Figure 6F).

Chemogenetic manipulation of LSv GABAergic neurons or LSv^GABA^–PVN projections did not significantly affect despair-like behavioral performance in the TST (Figures 2I,3G,5I,6G). These results appear inconsistent with our previous findings, which indicated that LSv plays an essential role in stress vulnerability (Wang et al., 2022). Differences in manipulation strategies (pharmacological vs. chemogenetical) may account for this discrepancy.

Overall, this study identified that the LSv^GABA^–PVN pathway plays some roles in anxiety-like behavioral performance in mice. The limitations of this study are worth noting to avoid overstating the results. First, only male mice were used in our study, and whether similar effects and mechanisms are suitable to females is unclear. Second, although a majority of virus-infecting cells are GABAergic in chemogenetic studies, other kinds of cells are also infected, which may confound the results. GAD-cre transgenic animals along with a more precise cell phenotyping with high-resolution confocal microscopy are needed to further address this concern. Thirdly, in CSDS studies (Figures 3, 6), saline or CNO were administered daily for 10 days in different animals. Therefore, the results obtained sometimes cannot be directly compared with the results of baseline studies (Figures 2, 5) in which the behavioral performances were tested after a single acute injection of saline or CNO in the same animal at different times. All in all, we found LS GABAergic neurons, particularly the LS GABAergic–PVN circuit, has a regulatory role in pathological anxiety and is thus a potential therapeutic target for the treatment of emotional disorders.

## Data availability statement

The data in this manuscript had been deposited in Mendeley Data, https://data.mendeley.com/datasets/3kjtrsy5rz/1.

## Ethics statement

The animal study was approved by Animal Care and Use Committee of Nanyang Normal University. The study was conducted in accordance with the local legislation and institutional requirements.

## Author contributions

Y-JL: Conceptualization, Funding acquisition, Investigation, Methodology, Writing – original draft. YW: Data curation, Formal analysis, Project administration, Writing – review & editing. J-WW: Investigation, Supervision, Validation, Visualization, Writing – review & editing. JZ: Data curation, Investigation, Validation, Visualization, Writing – review & editing. B-LS: Investigation, Project administration, Validation, Writing – review & editing. YJ: Investigation, Project administration, Supervision, Writing – review & editing. L-FL: Conceptualization, Funding acquisition, Supervision, Writing – review & editing.
