# Supplementary material for: GABAergic synapses from the ventral lateral septum to the paraventricular nucleus of hypothalamus modulate anxiety
Source: Front Neurosci. 2024 Mar 19;18:1337207. doi: 10.3389/fnins.2024.1337207 (PMC10985145; doi:10.3389/fnins.2024.1337207)
Supplement: Supplementary file 1 [file Data_Sheet_1.docx]

**SUPPLEMENTARY 1. QUANTIFICATION AND STATISTICAL ANALYSIS**

**Figure 1**

Figure 1B. Independent samples t-test and Bayesian independent samples t-test, two tailed. t_(12)_= 16.504, *P* < 0.01, Cohen's d = 8.822, BF_+0_ = 3.667×10^6^.

**Figure 2. Two-way repeated measures ANOVA along with two-way Bayesian repeated measures ANOVA. ‘Group’ as between subject factor (CTR, Gq, Gi) and ‘Treatment’ as repeated factor (Saline, CNO).**

Figure 2D. Treatment: F_(1, 33)_ = 0.036, *P* = 0.851, *η*² = 3.676 × 10^-4^, BF_(incl)_ = 0.241. Group: F_(2, 33)_ = 1.009, *P* = 0.376, *η*² = 0.038, BF_(incl)_ = 0.382. Treatment × Group: F_(2, 33)_ = 0.227, *P* = 0.798, *η*² = 0.005, BF_(incl)_ = 0.231.

Figure 2E. Treatment: F_(1, 33)_ = 0.481, *P* = 0.493, *η*² = 0.005, BF_(incl)_ = 0.302. Group: F_(2, 33)_ = 1.690, *P* = 0.2, *η*² = 0.059, BF_(incl)_ = 0.525. Treatment × Group: F_(2, 33)_ = 0.067, *P* = 0.935, *η*² = 0.001, BF_(incl)_ = 0.206.

Figure 2F. Treatment: F_(1, 33)_ = 0.048, *P* = 0.828, *η*² = 5.236×10^-4^, BF_(incl)_ = 0.241. Group: F_(2, 33)_ = 2.077, *P* = 0.141, *η*² = 0.007, BF_(incl)_ = 0.670. Treatment × Group: F_(2, 33)_ = 0.336, *P* = 0.717, *η*² = 0.007, BF_(incl)_ = 0.071.

Figure 2G. Treatment: F_(1, 33)_ = 0.113, *P* = 0.738, *η*² = 0.001, BF_(incl)_ = 0.255. Group: F_(2, 33)_ = 1.406, *P* = 0.259, *η*² = 0.045, BF_(incl)_ = 0.398. Treatment × Group: F_(2, 33)_ = 0.123, *P* = 0.885, *η*² = 0.003, BF_(incl)_ = 0.239.

Figure 2H. Treatment: F_(1, 33)_ = 1.667, *P* = 0.206, *η*² = 0.023, BF_(incl)_ = 0.561. Group: F_(2, 33)_ = 0.114, *P* = 0.893, *η*² = 0.003, BF_(incl)_ = 0.175. Treatment × Group: F_(2, 33)_ = 0.872, *P* = 0.427, *η*² = 0.024, BF_(incl)_ = 0.401.

Figure 2I. Treatment: F_(1, 33)_ = 1.922, *P* = 0.175, *η*² = 0.020, BF_(incl)_ = 0.563. Group: F_(2, 33)_ = 0.872, *P* = 0.428, *η*² = 0.030, BF_(incl)_ = 0.312. Treatment × Group: F_(2, 33)_ = 1.534, *P* = 0.231, *η*² = 0.032, BF_(incl)_ = 0.578.

**Figure 3. Two-way ANOVA along with two-way Bayesian ANOVA. Group factor: CTR, Gq, Gi; treatment factor: vehicle, CNO.**

Figure 3B. Treatment: F_(1, 30)_ = 0.136, *P* = 0.714, *η*² = 0.004, BF_(incl)_ = 0.338. Group: F_(2, 30)_ = 1.102, *P* = 0.345, *η*² = 0.066, BF_(incl)_ = 0.408. Treatment × Group: F_(2, 30)_ = 0.586, *P* = 0.563, *η*² = 0.035, BF_(incl)_ = 0.402.

Figure 3C. Treatment: F_(1, 30)_ = 0.029, *P* = 0.866, *η*² = 9.402×10^-4^, BF_(incl)_ = 0.325. Group: F_(2, 30)_ = 0.081, *P* = 0.922, *η*² = 0.005, BF_(incl)_ = 0.200. Treatment × Group: F_(2, 30)_ = 0.347, *P* = 0.709, *η*² = 0.022, BF_(incl)_ = 0.343.

Figure 3D. Treatment: F_(1, 30)_ = 0.558, *P* = 0.461, *η*² = 0.018, BF_(incl)_ = 0.408. Group: F_(2, 30)_ = 0.026, *P* = 0.975, *η*² = 0.002, BF_(incl)_ = 0.194. Treatment × Group: F_(2, 30)_ = 0.621, *P* = 0.544, *η*² = 0.039, BF_(incl)_ = 0.392.

Figure 3E. Treatment: F_(1, 30)_ = 0.288, *P* = 0.596, *η*² = 0.009, BF_(incl)_ = 0.361. Group: F_(2, 30)_ = 0.391, *P* = 0.680, *η*² = 0.024, BF_(incl)_ = 0.248. Treatment × Group: F_(2, 30)_ = 0.599, *P* = 0.556, *η*² = 0.037, BF_(incl)_ = 0.395.

Figure 3F. Treatment: F_(1, 30)_ = 4.535, *P* = 0.042, *η*² = 0.087, BF_(incl)_ = 1.140. Group: F_(2, 30)_ = 2.631, *P* = 0.089, *η*² = 0.100, BF_(incl)_ = 0.667. Treatment × Group: F_(2, 30)_ = 6.289, *P* = 0.005, *η*² = 0.240, BF_(incl)_ = 9.279; Gq_VEH *vs*. Gq_CNO, *P* = 0.017.

Figure 3G. Treatment: F_(1, 30)_ = 1.241, *P* = 0.274, *η*² = 0.035, BF_(incl)_ = 0.531. Group: F_(2, 30)_ = 2.247, *P* = 0.123, *η*² = 0.126, BF_(incl)_ = 0.910. Treatment × Group: F_(2, 30)_ = 0.035, *P* = 0.966, *η*² = 0.002, BF_(incl)_ = 0.281.

**Figure 5. Two-way repeated measures ANOVA along with two-way Bayesian repeated measures ANOVA. ‘Group’ as between subject factor (CTR, Gq, Gi) and ‘Treatment’ as repeated factor (Saline, CNO).**

Figure 5D. Treatment: F_(1, 31)_ = 5.598, *P* = 0.024, *η*² = 0.041, BF_(incl)_ = 0.712. Group: F_(2, 31)_ = 6.578, *P* = 0.004, *η*² = 0.146, BF_(incl)_ = 3.257; Gq vs. Gi, P = 0.004. Treatment × Group: F_(2, 31)_ = 16.761, *P* < 0.01, *η*² = 0.244, BF_(incl)_ = 12660.955; Gi_VEH *vs.* Gi_CNO, *P* < 0.01; Gi_CNO vs. Gq_CNO, *P* < 0.01; Gi_CNO vs. CTR_CNO, P = 0.069.

Figure 5E. Treatment: F_(1, 31)_ = 2.307, *P* = 0.139, *η*² = 0.022, BF_(incl)_ = 0.649. Group: F_(2, 31)_ = 2.226, *P* = 0.125, *η*² = 0.086, BF_(incl)_ = 0.803. Treatment × Group: F_(2, 31)_ = 0.327, *P* = 0.724, *η*² = 0.006, BF_(incl)_ = 0.259.

Figure 5F. Treatment: F_(1, 31)_ = 0.056, *P* = 0.814, *η*² = 8.110×10^-4^, BF_(incl)_ = 0.249. Group: F_(2, 31)_ = 1.198, *P* = 0.315, *η*² = 0.037, BF_(incl)_ = 0.324. Treatment × Group: F_(2, 31)_ = 1.347, *P* = 0.275, *η*² = 0.039, BF_(incl)_ = 0.616.

Figure 5G. Treatment: F_(1, 31)_ = 0.003, *P* = 0.960, *η*² = 4.024×10^-5^, BF_(incl)_ = 0.249. Group: F_(2, 31)_ = 2.048, *P* = 0.146, *η*² = 0.050, BF_(incl)_ = 0.414. Treatment × Group: F_(2, 31)_ = 2.286, *P* = 0.119, *η*² = 0.073, BF_(incl)_ = 1.5.

Figure 5H. Treatment: F_(1, 31)_ = 18.257, *P* < 0.001, *η*² = 0.218, BF_(incl)_ = 3510.247; VEH vs. CNO, *P* < 0001 (Gq: VEH vs. CNO, *P* = 0.01; Gi: VEH vs. CNO, *P* = 1; CTR: VEH vs. CNO, *P* = 0.604). Group: F_(2, 31)_ = 8.465, *P* = 0.001, *η*² = 0.136, BF_(incl)_ = 5.755; CTR vs. Gq, *P* < 0.001. Treatment × Group: F_(2, 31)_ = 1.102, *P* = 0.345, *η*² = 0.026, BF_(incl)_ = 0.638.

Figure 5I. Treatment: F_(1, 31)_ = 0.240, *P* = 0.628, *η*² = 0.003, BF_(incl)_ = 0.285. Group: F_(2, 31)_ = 1.004, *P* = 0.378, *η*² = 0.030, BF_(incl)_ = 0.288. Treatment × Group: F_(2, 31)_ = 5.869, *P* = 0.007, *η*² = 0.137, BF_(incl)_ = 20.151. Gq_VEH *vs*. gCNO, P = 0.057.

**Figure 6. Two-way ANOVA along with two-way Bayesian ANOVA. Group factor: non-stress, CSDS stress; treatment factor: vehicle, CNO.**

Figure 6B. Group: F_(2, 30)_ = 6.716, *P* = 0.004, *η*² = 0.212, BF_(incl)_ = 5.904; Gq vs. CTR, *P* = 0.008; Gq vs. Gi, *P* = 0.014. Treatment: F_(1, 30)_ = 12.171, *P* = 0.002, *η*² = 0.212, BF_(incl)_ = 10.844; VEH vs. CNO, *P* = 0.002. Treatment × Group: F_(2, 30)_ = 3.952, *P* = 0.030, *η*² = 0.124, BF_(incl)_ = 2.376.

Figure 6C. Group: F_(2, 30)_ = 1.938, *P* = 0.162, *η*² = 0.107, BF_(incl)_ = 0.706. Treatment: F_(1, 30)_ = 1.665, *P* = 0.207, *η*² = 0.046, BF_(incl)_ = 0.617. Treatment × Group: F_(2, 30)_ = 0.355, *P* = 0.704, *η*² = 0.020, BF_(incl)_ = 0.345.

Figure 6D. Group: F_(2, 30)_ = 4.788, *P* = 0.016, *η*² = 0.187, BF_(incl)_ = 2.229. Treatment: F_(1, 30)_ = 1.806, *P* = 0.189, *η*² = 0.035, BF_(incl)_ = 0.549. Treatment × Group: F_(2, 30)_ = 4.941, *P* = 0.014, *η*² = 0.193, BF_(incl)_ = 4.312; Gq: VEH vs. CNO, *P* = 1; Gi: VEH vs. CNO, *P* = 0.041; CTR: VEH vs. CNO, *P* = 1.

Figure 6E. Group: F_(2, 30)_ = 0.227, *P* = 0.798, *η*² = 0.014, BF_(incl)_ = 0.221. Treatment: F_(1, 30)_ = 1.444, *P* = 0.239, *η*² = 0.044, BF_(incl)_ = 0.589. Treatment × Group: F_(2, 30)_ = 0.442, *P* = 0.647, *η*² = 0.027, BF_(incl)_ = 0.363.

Figure 6F. Group: F_(2, 30)_ = 11.038, *P* < 0.001, *η*² = 0.311, BF_(incl)_ = 23.957; Gi vs. CTR and Gi vs. Gq all P < 0.01. Treatment: F_(1, 30)_ = 5.902, *P* = 0.021, *η*² = 0.083, BF_(incl)_ = 1.588. Treatment × Group: F_(2, 30)_ = 6.466, *P* = 0.005, *η*² = 0.182, BF_(incl)_ = 8.722; Gq: VEH *vs.* CNO, *P* = 0.004.

Figure 6G. Group: F_(2, 30)_ = 0.081, *P* = 0.923, *η*² = 0.005, BF_(incl)_ = 0.202. Treatment: F_(1, 30)_ = 0.041, *P* = 0.841, *η*² = 0.001, BF_(incl)_ = 0.328. Treatment × Group: F_(2, 30)_ = 0.094, *P* = 0.910, *η*² = 0.006, BF_(incl)_ = 0.333.

**Figure supplement 1D.** One-way ANOVA along with Bayesian ANOVA. F_(2,17)_ = 128.827, *P* < 0.01, *η*² = 0.945, BF_(incl)_ = 1.6 × 10^7^; Gq *vs.* virus control and Gi *vs.* virus control, all *P* < 0.01.

**Figure supplement 2. Two-way ANOVA along with two-way Bayesian ANOVA. Group factor: non-stress, CSDS stress; treatment factor: vehicle, CNO.**

Figure supplement 2B. Group: F_(1, 20)_ = 13.533, *P* = 0.001, *η*² = 0.363, BF_(incl)_ = 21.624; CSDS stress *vs*. non-stress, P < 0.001. Treatment: F_(1, 20)_ = 2.682, *P* = 0.117, *η*² = 0.072, BF_(incl)_ = 0.925. Treatment × Group: F_(1, 20)_ = 1.053, *P* = 0.317, *η*² = 0.028, BF_(incl)_ = 0.599.

Figure supplement 2C. Group: F_(1, 20)_ = 5.373, *P* = 0.031, *η*² = 0.196, BF_(incl)_ = 2.306. Treatment: F_(1, 20)_ = 0.177, *P* = 0.679, *η*² = 0.006, BF_(incl)_ = 0.387. Treatment × Group: F_(1, 20)_ = 1.911, *P* = 0.182, *η*² = 0.070, BF_(incl)_ = 0.878.

Figure supplement 2D. Group: F_(1, 20)_ = 3.601, *P* = 0.072, *η*² = 0.141, BF_(incl)_ = 1.335. Treatment: F_(1, 20)_ = 0.694, *P* = 0.415, *η*² = 0.027, BF_(incl)_ = 0.474. Treatment × Group: F_(1, 20)_ = 1.165, *P* = 0.293, *η*² = 0.046, BF_(incl)_ = 0.672.

Figure supplement 2E. Group: F_(1, 20)_ = 0.014, *P* = 0.906, *η*² = 6.317×10^-4^, BF_(incl)_ = 0.376. Treatment: F_(1, 20)_ = 0.694, *P* = 0.415, *η*² = 0.031, BF_(incl)_ = 0.483. Treatment × Group: F_(1, 20)_ = 1.714, *P* = 0.205, *η*² = 0.076, BF_(incl)_ = 0.823.

Figure supplement 2F. Group: F_(1, 20)_ = 29.843, *P* < 0.001, *η*² = 0.559, BF_(incl)_ = 845.884; CSDS stress *vs*. non-stress, *P* < 0.001. Treatment: F_(1, 20)_ = 3.434, *P* = 0.079, *η*² = 0.064, BF_(incl)_ = 1.229. Treatment × Group: F_(1, 20)_ = 0.130, *P* = 0.722, *η*² = 0.002, BF_(incl)_ = 0.470.

Figure supplement 2G. Group: F_(1, 20)_ = 4.271, *P* = 0.052, *η*² = 0.173, BF_(incl)_ = 1.818. Treatment: F_(1, 20)_ = 0.037, *P* = 0.850, *η*² = 0.001, BF_(incl)_ = 0.379. Treatment × Group: F_(1, 20)_ = 0.447, *P* = 0.512, *η*² = 0.018, BF_(incl)_ = 0.534.

Figure supplement 2H. Group: F_(1, 20)_ = 20.089, *P* < 0.001, *η*² = 0.494, BF_(incl)_ = 166.947; CSDS stress *vs*. non-stress, P < 0.001. Treatment: F_(1, 20)_ = 0.210, *P* = 0.652, *η*² = 0.005, BF_(incl)_ = 0.408. Treatment × Group: F_(1, 20)_ = 0.375, *P* = 0.547, *η*² = 0.009, BF_(incl)_ = 0.513.

Figure supplement 2I. Group: F_(1, 20)_ = 1.155, *P =* 0.295, *η*² = 0.052, BF_(incl)_ = 0.573. Treatment: F_(1, 20)_ = 0.512, *P* = 0.483, *η*² = 0.023, BF_(incl)_ = 0.448. Treatment × Group: F_(1, 20)_ = 0.629, *P* = 0.437, *η*² = 0.028, BF_(incl)_ = 0.590.

Figure supplement 2J. Group: F_(1, 20)_ = 6.364, *P* = 0.02, *η*² = 0.238, BF_(incl)_ = 3.759; CSDS stress *vs*. non-stress, P = 0.02. Treatment: F_(1, 20)_ = 0.145, *P* = 0.708, *η*² = 0.005, BF_(incl)_ = 0.395. Treatment × Group: F_(1, 20)_ = 0.186, *P* = 0.670, *η*² = 0.007, BF_(incl)_ = 0.473.

Figure supplement 2K. Group: F_(1, 20)_ = 0.381, *P* = 0.544, *η*² = 0.018, BF_(incl)_ = 0.424. Treatment: F_(1, 20)_ = 0.857, *P* = 0.366, *η*² = 0.040, BF_(incl)_ = 0.513. Treatment × Group: F_(1, 20)_ = 0.095, *P* = 0.761, *η*² = 0.004, BF_(incl)_ = 0.517.

Figure supplement 2L. Group: F_(1, 20)_ = 4.375, *P* = 0.049, *η*² = 0.170, BF_(incl)_ = 1.749. Treatment: F_(1, 20)_ = 8.803×10^-4^, *P* = 0.977, *η*² = 3.419×10^-5^, BF_(incl)_ = 0.364. Treatment × Group: F_(1, 20)_ = 1.373, *P* = 0.255, *η*² = 0.053, BF_(incl)_ = 0.743.

Figure supplement 2M. Group: F_(1, 20)_ = 5.858, *P* = 0.025, *η*² = 0.224, BF_(incl)_ = 3.182; CSDS stress *vs*. non-stress, P = 0.025. Treatment: F_(1, 20)_ = 0.144, *P* = 0.708, *η*² = 0.006, BF_(incl)_ = 0.396. Treatment × Group: F_(1, 20)_ = 0.178, *P* = 0.677, *η*² = 0.007, BF_(incl)_ = 0.471.

**Figure supplement 3D.** One-way ANOVA along with Bayesian ANOVA. F_(2, 12)_ = 14.341, *P* < 0.01, *η*² = 0.705, BF_(incl)_ = 44.854. Gq *vs*. Gi, *P* < 0.01; Gq *vs*. CTR, *P* = 0.042.
