# Supplementary material for: GABAergic synapses from the ventral lateral septum to the paraventricular nucleus of hypothalamus modulate anxiety
Source: Front Neurosci. 2024 Mar 19;18:1337207. doi: 10.3389/fnins.2024.1337207 (PMC10985145; doi:10.3389/fnins.2024.1337207)

**Supplementary Figures**

**Figure supplement 1. DREADDs expressions and functional verification for virus targeted LSv GABA neurons.** (A): Immunohistological image showing colocalization of DREADDs (mCherry, red) and GABA (Green) in the LSv. The right panels are amplified images in the left box which showing the mCherry, GABA and the colocalization of mCherry and GABA. (B): quantification of mCherry neurons colabeled with GABA (up pie), and GABA neurons colabeled with mCherry (down pie), *n* = 3 animals. (C & D): representative images and qualifications of c-fos expressions after CNO injections with DREADDs targeting LSv GABAergic neurons. Panel ‘D’ is analyzed by one-way ANOVA. Data are presented mean ± SE, *n* = 6 animals in each group; ^*^*P* < 0.05, ^**^*P* < 0.01. Scale bars = 100 µm.

**
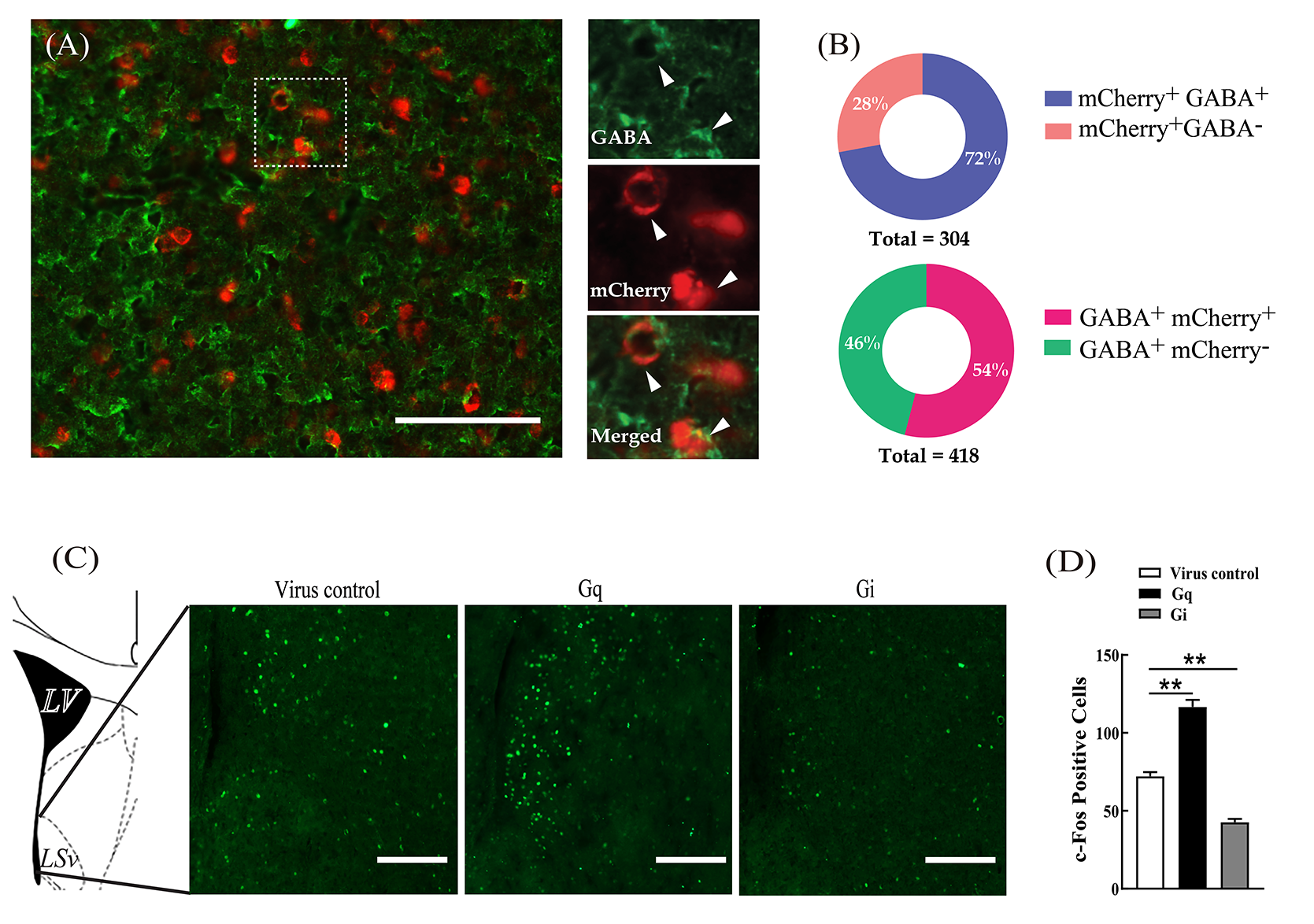
**

**Figure supplement 2. Behavioral performances of virus control animals under both non-stress and CSDS conditions.** (A): Timeline of experiments; (B-G): behavioral performances for virus expressed in the LSv GABAergic neurons in the OFT, EMP test, SI test and TST; (H-M): behavioral performances for virus expressed in the LSv^GABA^-PVN projections in the OFT, EMP test, SI test and TST. Data are presented as mean ± SE and analyzed by two-way ANOVA, *n* = 6 animals in each group; ^*^*P* < 0.05, ^**^*P* < 0.01. OFT: open-field test; EPM: elevated plus maze test; SI test: social interaction test; TST: tail suspension test; CTR: control; LSv: ventral part of the lateral septum; PVN: paraventricular nucleus of hypothalamus; CSDS: chronic social defeat stress.

**
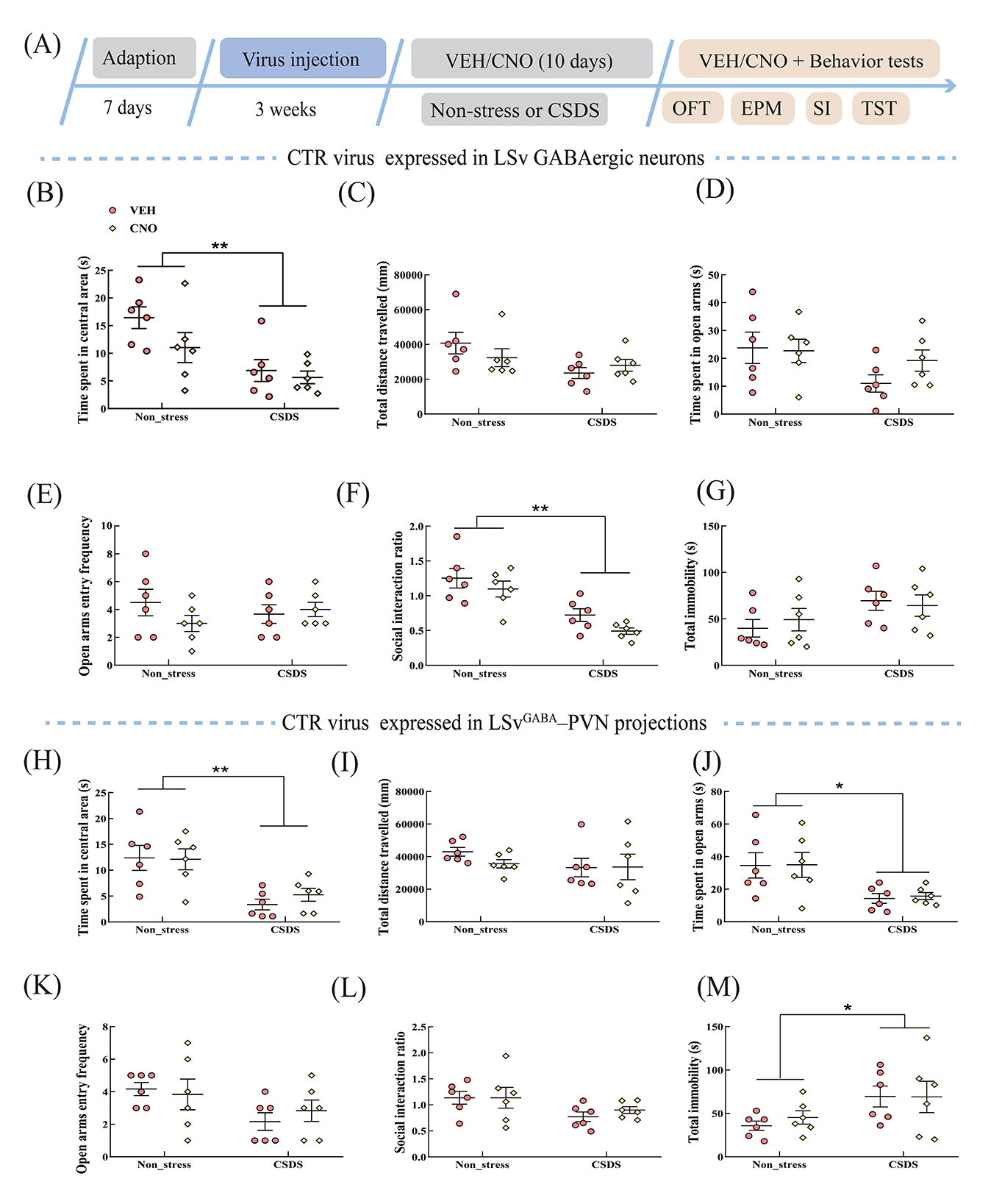
**

**Figure supplement 3. DREADDs expressions and functional examinations for virus expressed in LSv^GABA^–PVN circuit.** (A): Immunohistological image showing colocalization of DREADDs (mCherry, red) and GABA (Green) in the LSv. The right panels are amplified images in the left box which showing the mCherry, GABA and the colocalization of mCherry and GABA. (B): quantification of mCherry neurons colabeled with GABA (up pie), and GABA neurons colabeled with mCherry (down pie), *n* = 3. (C & D): representative images and qualifications of c-fos expressions in the PVN after CNO injections with DREADDs targeting LSv^GABA^–PVN circuit. Panel ‘D’ is analyzed by one-way ANOVA. Data are presented mean ± SE; *n* = 5 in each group; ^*^*P* < 0.05, ^**^*P* < 0.01. Scale bars = 100 µm.


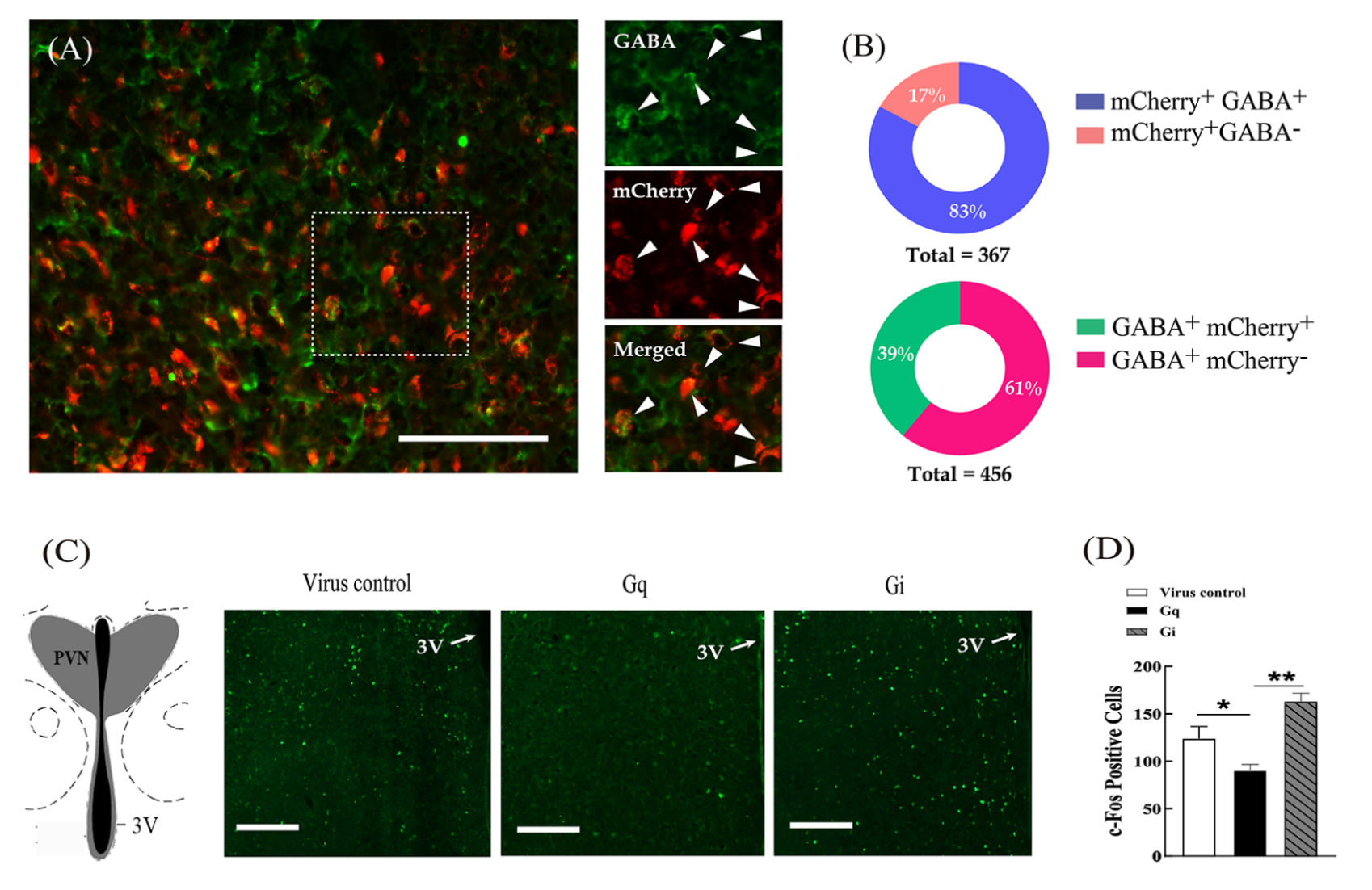

Supplement: Supplementary file 2 [file Data_Sheet_2.docx]
